# Etonogestrel Administration Reduces the Expression of PHOX2B and Its Target Genes in the Solitary Tract Nucleus

**DOI:** 10.3390/ijms23094816

**Published:** 2022-04-27

**Authors:** Silvia Cardani, Tara A. Janes, Jasmeen K. Saini, Simona Di Lascio, Roberta Benfante, Diego Fornasari, Silvia Pagliardini

**Affiliations:** 1 Department of Physiology, Faculty of Medicine and Dentistry, University of Alberta, Edmonton, AB T6G2E1, Canada; cardani@ualberta.ca (S.C.); tjanes@ualberta.ca (T.A.J.); 2 Department of Medical Biotechnology and Translational Medicine, Università degli Studi di Milano, Via Vanvitelli 32, 20129 Milan, Italy; simona.dilascio@unimi.it (S.D.L.); roberta.benfante@in.cnr.it (R.B.); 3 Women and Children’s Health Research Institute, University of Alberta, Edmonton, AB T6G2E1, Canada; jksaini@ualberta.ca; 4 Neuroscience and Mental Health Institute, University of Alberta, Edmonton, AB T6G2E1, Canada; 5 CNR-Institute of Neuroscience, Via Raoul Follereau 3, 20854 Vedano al Lambro, Italy; 6 NeuroMi-Milan Centre for Neuroscience, University of Milano Bicocca, 20129 Milan, Italy; 7 3-020F Katz Group Centre for Pharmacy and Health Research, Department of Physiology, University of Alberta, 11315 87 Ave NW, Edmonton, AB T6G2E1, Canada

**Keywords:** congenital central hypoventilation syndrome, chemoreflex response, breathing, transcription, autonomic nervous system, progesterone

## Abstract

Heterozygous mutations of the transcription factor *PHOX2B* are responsible for Congenital Central Hypoventilation Syndrome, a neurological disorder characterized by inadequate respiratory response to hypercapnia and life-threatening hypoventilation during sleep. Although no cure is currently available, it was suggested that a potent progestin drug provides partial recovery of chemoreflex response. Previous in vitro data show a direct molecular link between progestins and PHOX2B expression. However, the mechanism through which these drugs ameliorate breathing in vivo remains unknown. Here, we investigated the effects of chronic administration of the potent progestin drug Etonogestrel (ETO) on respiratory function and transcriptional activity in adult female rats. We assessed respiratory function with whole-body plethysmography and measured genomic changes in brain regions important for respiratory control. Our results show that ETO reduced metabolic activity, leading to an enhanced chemoreflex response and concurrent increased breathing cycle variability at rest. Furthermore, ETO-treated brains showed reduced mRNA and protein expression of PHOX2B and its target genes selectively in the dorsal vagal complex, while other areas were unaffected. Histological analysis suggests that changes occurred in the solitary tract nucleus (NTS). Thus, we propose that the NTS, rich in both progesterone receptors and PHOX2B, is a good candidate for ETO-induced respiratory modulation.

## 1. Introduction

Congenital central hypoventilation syndrome (CCHS) is a rare complex condition, characterized by impaired chemosensitivity and autonomic disfunction, which is caused by the genetic mutation of the transcription factor paired-like homeobox 2b (PHOX2B) [1,2,3,4]. PHOX2B is essential for the development of several classes of autonomic central and peripheral neurons [5,6,7,8]. While in many neurons its expression is reduced or silenced during postnatal development, several brainstem neurons maintain PHOX2B expression throughout life [9]. Among those are the neurons of the retrotrapezoid nucleus (RTN), a key hub for central chemoreflex response [10], and the dorsal vagal complex (DVC), a relay structure for multiple visceral, respiratory, and metabolic functions [11,12], including the nucleus of the solitary tract (NTS), the area postrema (AP) and the dorsal nucleus of the vagus (X) [9].

A clinical feature of CCHS is sleep-related hypoventilation, with severe cases also displaying hypoventilation during wakefulness [1,3,4], which has been suggested to be the consequence of improper development or function of PHOX2B-expressing neurons in the RTN [13,14], although other brain structures have been implicated from structural and functional magnetic resonance imaging studies in CCHS patients [15,16].

Respiratory stimulants commonly used in other respiratory disorders have proven ineffective in CCHS patients, and the only current options for maintaining ventilation (and survival) during sleep are mechanical ventilation or diaphragm pacing [4,17,18]. Interestingly, a serendipitous finding in two CCHS patients indicates potentiation of baseline respiratory frequency, reduced end-tidal CO_2_ [19] and partial recovery of CO_2_ chemosensitivity with the use of a potent progestin contraceptive, desogestrel [20].

Progesterone (PG) has been proposed to act as a respiratory stimulant [21,22], although studies investigating PG and hormone replacement therapy to counteract breathing disorders in menopausal women have produced mixed results [23,24,25,26,27,28,29,30]. Studies in experimental models have identified multiple sites and mechanisms of action of progesterone, its metabolites, and synthetic progestins due to the widespread expression of its receptors in the brain [31,32,33,34]. Central and peripheral areas potentially involved in respiratory potentiation by progestins are peripheral chemoreceptors in the carotid bodies, serotonergic neurons in the raphe nuclei, the solitary tract nucleus, the ventral tegmental area, the hypoglossal nucleus, the locus coeruleus, the parabrachial nucleus, and the hypothalamus [21,22,31,35,36,37]. Progestins may act through activation of both nuclear and membrane receptors to induce changes in gene expression, or via modulation of GABAergic, glutamatergic, nicotinic, serotonergic, and oxytocinergic receptors, in addition to modulation of various ion channels (reviewed in [21,31]).

Prior in vivo and in vitro studies in perinatal rodents suggest that desogestrel and its active metabolite, etonogestrel (3-ketodesogestrel; ETO) may have acute stimulatory effects on respiratory frequency in vivo and on the response to metabolic acidosis in vitro [19], possibly due to ETO stimulation of pontine and midbrain areas that are rich in PG receptors (PGR), such as locus coeruleus, hypothalamus or periaqueductal gray [38], in addition to the raphe nuclei [19].

Interestingly, both ETO and PG administration in neuroblastoma cell lines, expressing both PHOX2B and nuclear PGR, induced a direct reduction in PHOX2B expression, as well as reduced promoter activity of PHOX2B target genes [39]. In vitro and in vivo results, thus, suggest a potential functional interaction between ETO-induced PGR activation, PHOX2B expression and its transcriptional activity, and respiration.

The partial recovery of CO_2_ chemosensitivity with desogestrel in CCHS patients and the in vitro stimulatory ETO effect in perinatal rodents prompted us to investigate the effects of long-term systemic ETO administration in healthy adult female rats. We hypothesized that ETO would stimulate breathing during ventilatory challenges and based on cell culture studies, would induce changes in gene and protein targets expression that are dependent on PGR activation in the brain.

In this study, we tested baseline breathing and the chemoreflex response, following instrumentation of female rats with slices of Nexplanon^®^ rods (Merck, MSD Corp, Kenilworth, NJ, USA) to continuously deliver ETO over a four-week period. At the end of the treatment period, brain regions of interest were isolated, and protein and gene expression was evaluated. Our results indicate that while long-term ETO administration does not alter the absolute magnitude of chemoreflex responses, it results in a mismatch between ventilation and metabolism, such that CO_2_-responsiveness is enhanced. We also noted increased variability in the breath cycle period of ETO-treated rats, pointing to additional changes to respiratory control. Furthermore, while expression of genes and proteins of interest was not affected by ETO treatment in the hypothalamus, locus coeruleus and the parafacial region (including the RTN), we observed a reduction in gene and protein expression of PHOX2B, PHOX2A and known PHOX2B target genes in the dorsal vagal complex (DVC). Histological analysis verified that changes in PHOX2B expression occurred specifically in the nucleus of the solitary tract.

## 2. Results

### 2.1. Etonogestrel Serum Levels in Nexplanon^®^-Instrumented Rats

In women, Nexplanon^®^ treatment results in ~200–250 pg/mL ETO in the serum for a 3-year period [40,41]. In order to achieve comparable serum concentrations in our adult female rats, we tested variable lengths of Nexplanon^®^ rods in pilot studies and determined ETO serum levels 4 weeks after instrumentation. We found that 1000 μm-thick Nexplanon^®^ rods resulted in 236.8 ± 50.0 pg/mL ETO (*n* = 5), while thinner rods produced serum levels below our target (250 μm = 176.0 ± 39.2 pg/mL, *n* = 5; 500 μm = 175.3 ± 7.5 pg/mL, *n* = 4). Thus, 1000 μm Nexplanon^®^ rod lengths were used for the remainder of the study.

### 2.2. Etonogestrel Treatment Down-Regulates Phox2b Gene and Protein Expression in the Dorsal Vagal Complex

Since ETO administration in neuroblastoma cell lines affects the PHOX2B pathway through PGR activation [39], we investigated the cellular and molecular effects of ETO treatment within selected regions of the brain: the dorsomedial hypothalamus (DMH), locus coeruleus (LC), parafacial area (pF, including RTN and the facial nucleus, FN) and the DVC (including the nucleus of the solitary tract, NTS, area postrema, AP, and dorsal motor nucleus of the vagus, X). These regions were selected based on their central role in respiratory chemoreception and, with the exception of DMH, on the expression of both PHOX2B and PGR [37,42,43].

**Figure 1:**
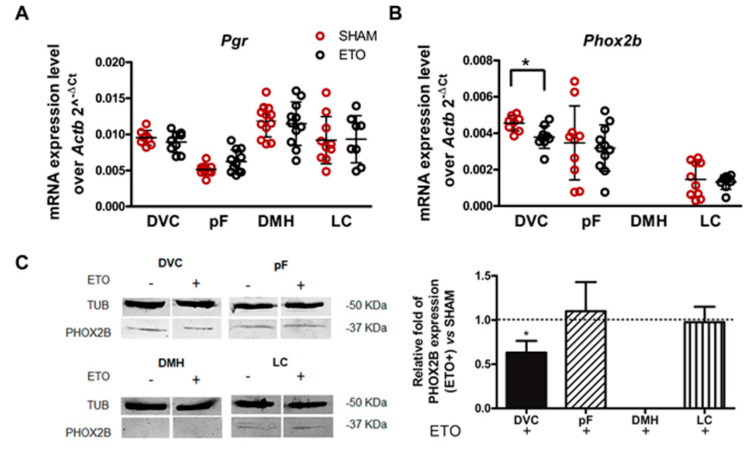
ETO reduces *Phox2b* mRNA and PHOX2B protein expression within DVC. (**A**,**B**) qPCR analysis of *Pgr* and *Phox2b* mRNA expression normalised to the endogenous standard *Actb* in naïve rats after 4 weeks ETO (black empty dots)/SHAM (red empty dots) treatment in DVC, pF, DMH and LC brain regions. Data are expressed as 2^−^^Δ^^Ct^ and each data point represents one rat to show between-sample variability: black horizontal lines correspond to the mean. (**C**) Representative Western blot analysis and quantification of the effect of ETO on PHOX2B protein expression in the same area. Left: 80 μg of protein extract from brain tissues from SHAM (ETO-) or treated (ETO+) rats, were size fractionated by means of SDS-PAGE and transferred to a nitrocellulose membrane and labelled for β-tubulin (TUB) and PHOX2B. Right: relative quantification of PHOX2B protein in ETO-treated rats. Results are reported as mean values (± SD) normalised to that of TUB in at least three independent experiments and are expressed as fold expression over SHAM rats (=1). * *p* < 0.05, Significant difference between SHAM and ETO-treated rats were assessed by Student’s *t*-test in each examined area.

To evaluate changes in gene expression, qPCR analysis was performed on mRNA isolated from these brain areas. No changes in *Pgr* mRNA expression between the control and ETO-treated groups were observed in any of the areas investigated (Figure 1A). Quantification of *Phox2b* mRNA showed that ETO treatment reduced *Phox2b* expression by 16.81 ± 0.14% selectively in the DVC (t_(15)_ = 2.923, *p* = 0.01, *n* = 9; Figure 1B). In contrast, the LC and the pF were not affected, whereas DMH did not express *Phox2b* in either group, as reported previously [9].

Western blot and densitometry analysis were also performed on the same tissue samples and confirmed significantly reduced PHOX2B protein levels in the DVC (39.01 ± 0.13% decrease, t_(6)_ = 3.464, *p* = 0.013, *n* = 3; Figure 1C). These results align with our previous findings in neuroblastoma cell lines, in which ETO treatment dramatically reduced PHOX2B protein levels, while *PHOX2B* mRNA levels were still reduced, but less affected [39].

**Figure 2:**
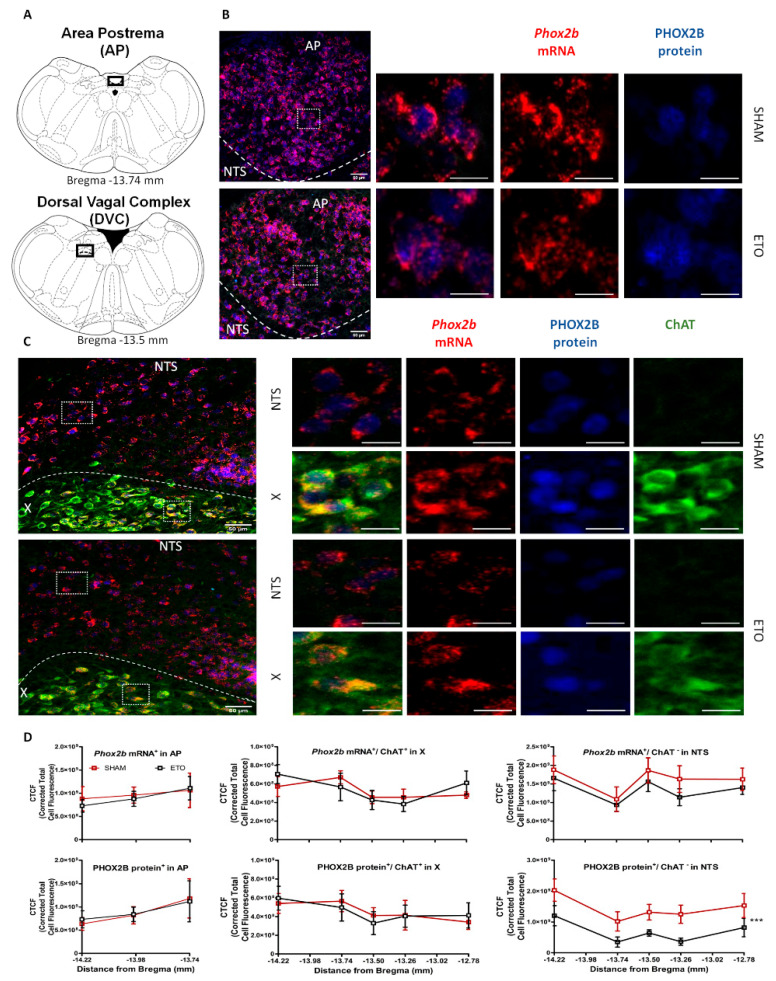
ETO reduces the expression of PHOX2B in NTS neurons. (**A**) Schematic and representative images of transverse brainstem sections at the level of the AP (top, −13.47 mm distance from Bregma) and the DVC (bottom, −13.50 mm distance from Bregma) showing the areas of interest illustrated in (**B**,**C**). (**B**,**C**) *Phox2b*^+^ mRNA (red) and PHOX2B (blue) and ChAT protein (green) expression in AP (**B**), NTS neurons and X motoneurons. (**C**) in SHAM (top) and ETO (bottom)-treated rats (magnified view insert). Scale bar = 50 µm. (**D**) Quantification of *Phox2b* mRNA (top) and protein (bottom) fluorescence staining intensity along the rostro-caudal extension of the AP, X and NTS in SHAM (red) and ETO (black) -treated rats. Mean corrected total cell fluorescence (CTCF) value ± SEM calculated from five different rats per group (see methods for details). Significant differences were observed in PHOX2B protein expression in NTS neurons (repeated-measures ANOVA, *** *p* < 0.001).

To further verify this reduction, at both mRNA and protein levels, and to investigate spatial localization within the DVC, we performed a semi-quantitative analysis of *Phox2b mRNA* expression with in situ hybridization (RNAScope) and PHOX2B protein expression with immunofluorescence staining (*n* = 5 rats per group). Representative images of the staining in the three areas of the DVC are shown in Figure 2. No significant differences were observed in the PHOX2B expression between SHAM and ETO-treated groups in the neurons in the AP (F_(1,10)_ = 0.939, *p* = 0.4; Figure 2D) and in ChAT^−^positive X motoneurons (F_(1,10)_ = 0.994, *p* = 0.46; Figure 2D), at different rostro-caudal levels of the medulla. Although the semi-quantitative analysis of *Phox2b* mRNA did not show significant reduction in the NTS neurons (−17.19 ± 0.32%, F_(1,10)_ = 1.851 *p* = 0.09; Figure 2D, top), a reduction in PHOX2B protein was observed (−52.81 ± 0.31%, F_(1,10)_ = 4.842, *p*= 0.0003, Figure 2D, bottom) across the rostro-caudal extension of the medulla.

These results suggest that ETO treatment resulted in down-regulation of the PHOX2B protein in the DVC, particularly in the NTS region.

### 2.3. ETO Treatment Does Not Affect PHOX2B Expression in RTN CO_2_-Sensing Neurons

**Figure 3:**
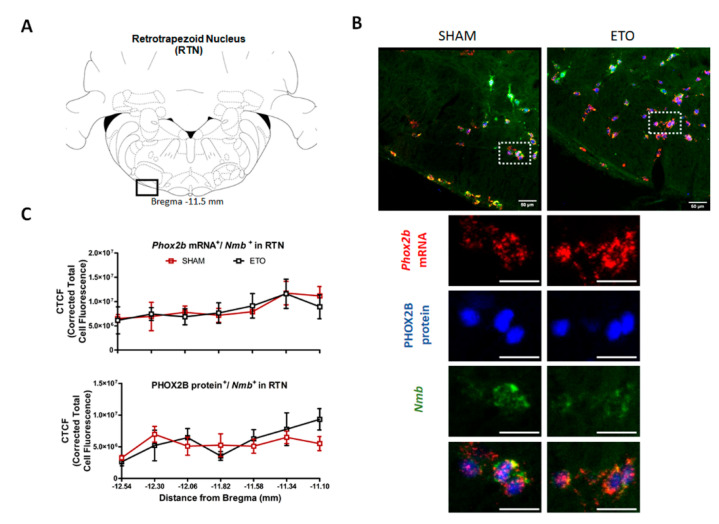
ETO does not affect expression of PHOX2B in RTN neurons. (**A**) Schematic and representative image of a transverse brainstem section at the level of the RTN (−11.5 mm distance from Bregma) showing. (**B**) RTN *Phox2b^+^/Nmb^+^* neurons in SHAM (left) and ETO (right) rats (magnified view insert). Scale bar = 50 µm. (**C**) Mean-corrected total cell fluorescence (CTCF) value ± SEM calculated from five different rats per group (see methods for details) along the rostro-caudal extension of the RTN. Mean CTCF value ± SEM combined from five different animals per group.

We further investigated whether ETO treatment altered the expression of PHOX2B in RTN neurons of healthy female rats (Figure 3). Representative images of *Phox2b^+^/Nmb^+^* RTN neurons used for the analysis are shown in Figure 3B. Consistent with our qPCR and Western blot data in the larger pF area (Figure 1), no significant changes in the expression of PHOX2B in *Phox2b^+^/Nmb^+^* cells were observed, both at the mRNA and protein levels, between SHAM- and ETO-treated rats (F_(1,10)_ = 0.983, *p* = 0.48; Figure 3C). Interestingly, the mRNA levels of both *Gpr4* and *TASK2*, two key pH sensors in RTN chemoreception, did not show significant changes between SHAM and ETO groups (*Gpr4* t_(20)_ = 1.495, *p* = 0.1505, *n* = 10; *TASK2*: t_(20)_ = 0.5942, *p* = 0.5591, *n* = 10). These results suggest that ETO does not affect the expression of PHOX2B, and possibly neuronal activity through gene transcription of its target genes within the pF area.

### 2.4. ETO-Mediated PHOX2B Down-Regulation in the NTS Results in Decreased Expression of PHOX2B Target Genes

**Figure 4:**
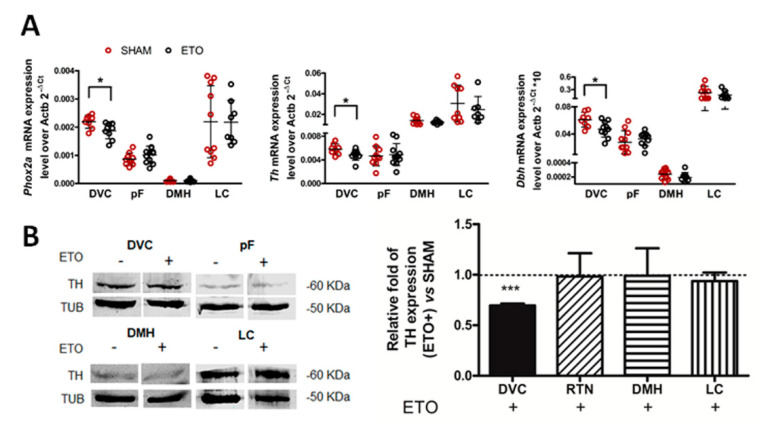
ETO reduces the expression of PHOX2B target genes in DVC. (**A**) qPCR analysis of *Phox2a*, *Th* and *Dbh* mRNA expression normalised to the endogenous standard *Actb* after 4 weeks ETO (black empty dots)/SHAM (red empty dots) treatment in the brain regions of interest. Data are expressed as 2^−^^Δ^^Ct^ and each data point represents one rat measurement; black horizontal lines correspond to the mean values. (**B**) Representative Western blot analysis and quantification of the effect of ETO on TH protein expression. Left: expression of TH and TUB in NTS, pF, DMH and LC in SHAM (ETO-) treated (ETO+) rats. Right: TH protein expression quantification relative to TUB in ETO-treated samples (black bars) relative to SHAM (mean values ± SD)., Significant differences in *Phox2a*, *Th* and *Dbh* mRNA expression and TH protein expression between SHAM and ETO-treated rats were observed only in DVC area (* *p* < 0.05 and *** *p* < 0.001, Student’s *t*-test).

In order to further investigate the relationship between ETO treatment and PHOX2B activity, we investigated whether reduced PHOX2B protein levels could, in turn, affect the expression of known PHOX2B target genes: *Phox2a*, Tyrosine hydroxylase (*Th*) and dopamine beta hydroxylase (*Dbh*) [44,45,46,47]. As in the case of *Phox2b*, a significant reduction in *Phox2a* (−14.81 ± 0.13%, t_(15)_ = 2.577, *p* = 0.02, *n* = 9), *Th* (−16.74 ± 0.15%, t_(15)_ = 2.253, *p* = 0.040, *n* = 9) and *Dbh* (−22.51 ± 0.19%, t_(16)_ = 2.541 *p* = 0.022, *n* = 9) mRNA levels were observed in ETO-treated rats in comparison to SHAM rats at the level of the DVC (Figure 4A). Of note, no significant changes were observed in the other areas considered, thus, supporting the direct effect of ETO on PHOX2B expression and its transcriptional activity, specifically at the level of DVC.

The reduction in TH expression was also confirmed at the protein level (30.45 ± 0.02% t_(4)_ = 15.53, *p* = 0.0001 *n* = 3; Figure 4B) in DVC, although we were unable to confirm the reduction in PHOX2A and DBH due to the low sensitivity of the antibodies used. In conclusion, our findings demonstrate that ETO directly affects PHOX2B and its target genes by reducing their expression, specifically within the DVC.

### 2.5. ETO Treatment Does Not Alter Baseline Breathing or Respiratory Response to Chemoreflex Activation

**Figure 5:**
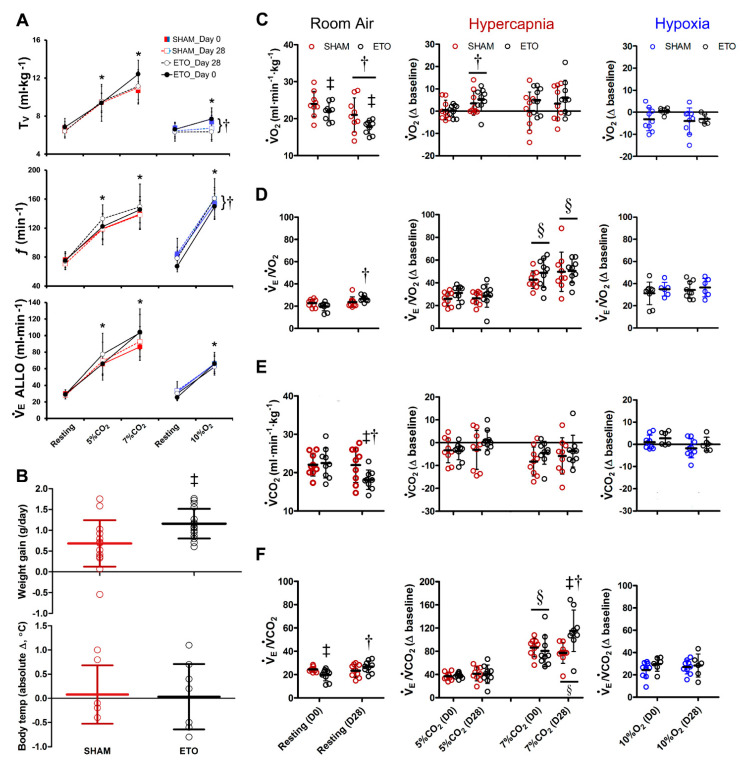
Etonogestrel (ETO) enhances the convective requirement ratio during hypercapnia. (**A**) Tidal volume (T_V_), breathing frequency (*f*) and allometric ventilation ($\overset{•}{V}$_E_ ALLO) showed expected increases during chemoreflex activation. ETO did not alter respiratory responses when measured under resting conditions or chemoreflex activation. However, hypoxia-induced T_V_ was blunted and *f* increased in both treatment groups post-surgery. (**B**) Rats treated with ETO for 28 days gained more weight vs. SHAM but resting body temperature was unchanged. (**C**–**F**) Metabolic measurements of O_2_ and CO_2_ production ($\overset{•}{V}$O_2_, $\overset{•}{V}$CO_2_) and expressed as a function of $\overset{•}{V}$_E_ ($\overset{•}{V}$_E_/$\overset{•}{V}$O_2_, $\overset{•}{V}$_E_/$\overset{•}{V}$CO_2_). Data were analysed during room air (left panels; absolute data) hypercapnia (middle panel, absolute change from room air) and hypoxia (right panel, absolute change from room air). Chemoreflex activation led to expected increases in $\overset{•}{V}$_E_/$\overset{•}{V}$O_2_ and $\overset{•}{V}$_E_/$\overset{•}{V}$CO_2_ compared to resting conditions. ETO treatment was significantly increased for resting $\overset{•}{V}$O_2_, $\overset{•}{V}$CO_2_ and the hypercapnic response, but not the hypoxic response. * Different from corresponding baseline; † different from corresponding pre-surgery value; ‡ different from SHAM; § different from 5% CO_2_; *p* < 0.05.

Based on the observed ETO-induced changes in gene and protein expression in the DVC, a major respiratory control centre, we questioned whether respiratory behaviour was altered in freely behaving ETO-treated rats. When rats were tested inside a whole-body plethysmograph in room air (i.e., resting conditions, *n* = 9 rats per group), there was no effect of surgery or ETO-treatment on the tidal volume (T_V_), respiratory frequency (*f*), or allometric minute ventilation ($\overset{•}{V}$_E_ ALLO) during room air recordings (Figure 5A).

Hypercapnia produced dose-dependent increases in T_V_ (5% CO_2_: 38 ± 19%, 7% CO_2_: 82 ± 17%; *p* < 0.01, Bonferroni) and *f* (5%: 63 ± 10%, 7%: 94 ± 17%; *p* < 0.05; Bonferroni) in pre-surgical recordings of ETO rats (ETO_Day 0). This led to an increased $\overset{•}{V}$_E_ ALLO of 127 ± 38% in 5% CO_2_ and 257 ± 54% in 7% CO_2_, as compared to restful breathing (*p* < 0.001, Bonferroni; Figure 5A). Similar relationships were observed in ETO rats 28-days post-surgery (vs. corresponding pre-surgery value: *p* = 1.0, Bonferroni). No significant differences in T_V_, *f* or $\overset{•}{V}$_E_ were observed between SHAM and ETO-treated rats (T_V_; F_(1,16)_ = 0.788, *p* = 0.39; *f*: F_(1,16)_ = 1.38, *p* = 0.26; $\overset{•}{V}$_E_ ALLO: F_(1,16_) = 2.17, *p* = 0.16).

Hypoxia increased steady state T_V_ (17 ± 11%, *p* = 0.03, Bonferroni), *f* (124 ± 30%, *p* < 0.001, Bonferroni) and $\overset{•}{V}$_E_ ALLO (161 ± 41%, *p* < 0.001, Bonferroni) in ETO rat pre-surgical recordings (Day 0, Figure 5A). Similar respiratory responses during chemoreflex stimulation were observed pre-surgery in the SHAM cohort (treatment effect: Tv; F_(1,15)_ = 0.36, *p* = 0.56; *f*: F_(1,15)_ = 0.57, *p* = 0.46; $\overset{•}{V}$_E_ ALLO: F_(1,15)_ = 1.37, *p* = 0.26). The ventilatory pattern response to hypoxia was altered in both SHAM and ETO groups 28-days post-surgery: hypoxia-induced increases in T_V_ were blunted (*p* = 0.013, Bonferroni) while *f* response was enhanced (*p* = 0.024, Bonferroni). However, $\overset{•}{V}$_E_ ALLO was unaffected (*p* = 1.0, Bonferroni).

### 2.6. ETO Enhances Alveolar Hyperventilation during High CO_2_

Body weight and resting body temperatures were similar between treatment groups prior to surgery (ETO: 309 ± 25 g, 37.4 ± 0.5 °C; SHAM: 324 ± 36 g, 37.1 ± 0.3 °C). Etonogestrel treatment led to significantly more weight gain as compared to SHAM rats (*p* = 0.008), while resting body temperature was unchanged (*p* = 0.055; Figure 5B). Since female sex hormone supplementation is associated with weight gain and lower metabolism [48], we next determined if ETO treatment affected metabolism and ventilatory–metabolic relationships in our rat model using pull-mode indirect calorimetry.

During room air, oxygen consumption ($\overset{•}{V}$O_2_) was reduced in both treatment groups 28-days post-surgery (F(_1,16)_ = 11.96, *p* = 0.003) and this effect was more important in the ETO-treated group compared to SHAM rats (treatment effect: F_(1,16)_ = 5.64, *p* = 0.03; Figure 5C). Analysis of the oxygen convective requirement ratio shows that $\overset{•}{V}$e/$\overset{•}{V}$O_2_ was increased following 28 days of ETO treatment (41 ± 28%; *p* < 0.001, Bonferroni; Figure 5D) because of the reduction in O_2_ consumption relative to ventilation. Carbon dioxide production was also reduced in ETO rats relative to pre-surgical (Day 0) baseline (*p* < 0.001, Bonferroni) and was different from post-surgical SHAM (*p* = 0.043, Bonferroni; Figure 5E). Treatment with ETO increased $\overset{•}{V}$e/$\overset{•}{V}$CO_2_ 28-days post-surgery (40 ± 25%; *p* < 0.001, Bonferroni; Figure 5F). These data suggest reduced metabolism relative to ventilation in both treatment groups at 28-days post-surgery and may result from increased body mass, particularly in ETO-treated rats.

In terms of chemoreflex responses, the absolute change in hypercapnia vs. room air for $\overset{•}{V}$O_2_ measured at 28 days was increased compared to day 0, in both SHAM and ETO-treated rats (*p* = 0.04, Bonferroni). The absolute change vs. room air for $\overset{•}{V}$CO_2_ was not affected by hypercapnia exposure in either treatment group. As expected, both $\overset{•}{V}$e/$\overset{•}{V}$O_2_ and $\overset{•}{V}$e/$\overset{•}{V}$CO_2_ of ETO- and SHAM-treated rats increased during 5 and 7% CO_2_ exposure (Figure 5D,F). The $\overset{•}{V}$e/$\overset{•}{V}$O_2_ and $\overset{•}{V}$e/$\overset{•}{V}$CO_2_ during 7% CO_2_ were greater than those of 5% CO_2_ for both groups (*p* ≤ 0.01, Bonferroni). Interestingly, ETO treatment significantly increased $\overset{•}{V}$e/$\overset{•}{V}$CO_2_ during 7% CO_2,_ as compared to both pre-surgery (pre: Δ81 ± 27; post: Δ115 ± 36; *p* < 0.001, Bonferroni) and SHAM rats (SHAM post: Δ77 ± 18; *p* = 0.009, Bonferroni). The increased $\overset{•}{V}$e/$\overset{•}{V}$CO_2_ during 7% CO_2_ observed for ETO-treated rats may be ascribed to the significantly decreased room air $\overset{•}{V}$CO_2_ for this group (Figure 5E), combined with further decreases in absolute $\overset{•}{V}$CO_2_ in 7% CO_2_ at day 28 vs. day 0 (*p* = 0.01, Bonferroni, absolute $\overset{•}{V}$CO_2_ day 0 = 19.1 ± 4.7; day 28 = 12.9 ± 6.5) and slightly increased minute ventilation. These data suggest that ETO treatment enhances the hyperventilatory response to high CO_2_.

Hypoxia did not change $\overset{•}{V}$O_2_ or $\overset{•}{V}$CO_2,_ as compared to baseline; however, $\overset{•}{V}$e/$\overset{•}{V}$O_2_ and $\overset{•}{V}$e/$\overset{•}{V}$CO_2_ were increased relative to room air, indicating expected increases in ventilation. No significant differences in metabolic measurements were observed between ETO-treated rats and SHAM controls in hypoxia ($\overset{•}{V}$e/$\overset{•}{V}$O_2_: F_(1,13)_ = 1.32, *p* = 0.27; $\overset{•}{V}$e/$\overset{•}{V}$CO_2_: F_(1,13)_ = 1.41, *p* = 0.26).

### 2.7. ETO Increases Inter-Breath Variability

**Figure 6:**
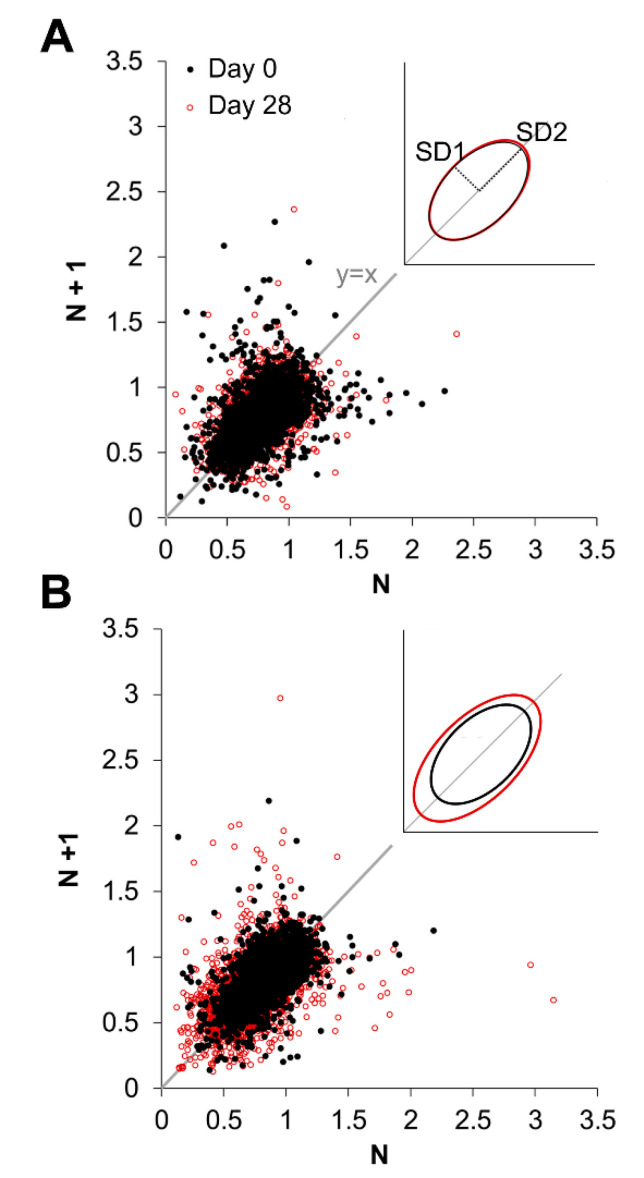
Etonogestrel (ETO) treatment increases breath cycle variability. Poincaré plots showing breath cycle duration at baseline (black) and 4 weeks following SHAM surgery (**A**), red or ETO-treatment (**B**) red. Inset, ellipses fit to the data based on measures of SD1 and SD2 (see methods and Table 1).

Changes in the convective requirement ratio, $\overset{•}{V}$e/$\overset{•}{V}$CO_2_, noted for room air and 7% CO_2_ conditions in response to ETO treatment may indicate enhanced excitability or instability in the underlying respiratory control networks [49]. To test this concept, we measured breath cycle period (inspiration to subsequent inspiration) during room air breathing and constructed Poincaré plots to visualize respiratory variability. These plots, shown in Figure 6, illustrate that the 4-week ETO treatment enhanced the inter-cycle variability, primarily by increasing the incidence of shortened cycles (i.e., data points closer to the origin). These data were quantified as standard metrics: SD1, SD2, SD1/SD2 and area, which define an ellipse representative of inter-cycle variability (standard deviation). Treatment with ETO increased SD2 (pre- vs. post-surgery *p* = 0.004; vs. SHAM *p* = 0.028, Bonferroni) and Area (pre- vs. post-surgery *p* = 0.003; vs. SHAM *p* = 0.021, Bonferroni; Table 1).

## 3. Discussion

This study investigated whether cellular and functional changes in respiratory control occur following administration of a potent progestin contraceptive drug, ETO. Because of the serendipitous observation that desogestrel improves ventilatory function in CCHS patients [19,20] and the respiratory potentiation observed in neonatal rodents following acute administration [19,38,50], we sought to investigate whether the active metabolite of desogestrel, ETO, may also have an effect on ventilation or gene expression in respiratory control areas, following chronic administration in adult female healthy rats. In our study, we chose to deliver ETO through a body-weight-appropriate Nexplanon^®^ rod section to obtain continuous ETO serum levels comparable to the ones reported in women using Nexplanon rods for contraceptive purposes [41] and avoid daily injections that could result in stress for the animals. We opted not to use an ovariectomized rat model because our objective was to assess whether ETO supplementation was effective compared to physiological, rather than hormonally impaired, conditions.

While ETO administration did not significantly affect T_v_, *f* and $\overset{•}{V}$e ALLO during baseline breathing, and hypoxic and hypercapnic chemoreflex responses in healthy female rats, the same ETO dose induced changes in metabolism (with consequent increased hyperventilatory response in hypercapnia), increased respiratory variability, and reduced gene and protein expression levels of PHOX2B and some of its target genes in the NTS, an important relay structure in cardiorespiratory and metabolic function.

ETO is a potent progestin of the gonane family, with a 25-fold greater affinity to PGRs than progesterone itself [32,40,51]. The biological effects are associated with genomic and non-genomic actions through PGRs, in addition to modulation of other neurotransmitters and channels in the brain [31,32,39,51,52]. Interestingly, recent work from our group has shown that in vitro, ETO down-regulates PHOX2B expression and its transcriptional activity. Specifically, ETO reduces the expression level of PHOX2B and its known target genes (T-cell leukaemia homeobox 2 and DBH) in neuroblastoma cell lines through activation of the nuclear PGR [39]. We, therefore, investigated whether this effect could also be observed in vivo, and we analysed different brain areas that are known to express PHOX2B and PGR and have a role in respiratory control. While ETO did not affect overall expression of PGR in any of the brain areas investigated, we observed a significant reduction in both mRNA and protein levels within the DVC. This reduction also affected known PHOX2B target genes that are present in this area, *Phox2a*, *Th* and *Dbh* [44,46,53]. Histological analysis verified that within the DVC dissected area, neurons in the AP and X were not apparently affected, but neurons in the NTS displayed a significant reduction in PHOX2B protein level. Surprisingly, the PHOX2B levels in the parafacial region, specifically in the *Nmb/Phox2b* -expressing neurons of the RTN, were not affected by the ETO treatment, an effect that may be explained by low levels of PGRs detected in these neurons.

The effects of progesterone and its synthetic derivatives on ventilation have been previously investigated, with various outcomes, depending on models used, dose, mode of administration, and sites of action [22,54,55,56,57,58,59,60]. Overall, there is general agreement that progesterone and its analogues act on various areas of the brain to potentiate ventilation, although the mechanistic details of these effects need further investigation [51,61].

The specific effects of ETO on breathing have been explored in neonatal rodents [19,38,50] and an increased respiratory frequency was observed in vivo and in vitro, possibly acting through modulation of GABAergic, glutamatergic and serotoninergic neurotransmission [19]. Furthermore, acute ETO administration in newborn rodents enhanced ventilation in vitro and ex vivo under metabolic acidosis and this effect was attributed to activation of supramedullary structures [38], possibly orexinergic neurons [50].

In contrast to perinatal data, we failed to observe any change in *f* and $\overset{•}{V}$_E_ ALLO in normoxia, hypoxia or hypercapnia in female rats chronically treated with ETO, although changes in respiratory variability were observed in normoxia. The differences in baseline breathing or chemoreflex response compared to previous studies may be due to different delivery methods, higher hormonal levels in mature rats compared to new-borns [62], different species, developmental changes in PGR expression [63,64,65], or distinct mechanisms of actions of ETO through development [22,66]. However, ventilatory effort at rest and during chemoreflex activation is driven, in part, by metabolic rates [67] and our data show that significant weight gain by ETO-treated rats was concurrent with reduced metabolism at rest (decreased $\overset{•}{V}$O_2_ and $\overset{•}{V}$CO_2_). Interestingly, ETO treatment increased the convective requirement ratio during 7% CO_2,_ suggesting a “mismatch” in respiratory control, whereby ventilatory effort was higher than predicted by metabolic rates [67]. Enhanced convective requirement ratios have been conceptualized by applying the concept of “loop-gain” to the sensitivity and efficiency of the neural, pulmonary and circulatory components of respiratory control to changing blood gases [49]. Based on our data, we propose that chronic ETO treatment enhances chemoreflex responsiveness to high CO_2_ (i.e., increased controller gain). While we cannot rule out changes in pulmonary or circulatory efficiency, the fact that ETO treatment enhanced responsiveness to high CO_2_, and not hypoxia, suggests changes specific to neural pathways governing the hypercapnic chemoreflex. Given that enhanced CO_2_ chemoreflex is associated with respiratory instability (such as periodic breathing [49,68]) and that blood CO_2_ (PCO_2_) plays a role in restful breathing [69], our data demonstrating increased breath cycle variability during room air in ETO-treated rats supports enhanced gain of the respiratory neural networks involved in CO_2_ sensing and integration.

Our molecular results in the NTS are particularly interesting, as this area has an important role in relaying multiple autonomic functions and integration of chemosensory stimuli [11,12], some of which are altered in CCHS pathology [70]. Although we cannot, at present, conclude that changes in expression of PHOX2B and its target genes in the NTS are responsible for the observed respiratory effects, these results raise the possibility that the NTS is the possible site of action of desogestrel in CCHS patients’ recovery [19,20]. Indeed, the NTS was originally proposed as a key site responsible for progesterone-induced ventilatory potentiation [56,57]. It is possible that the stimulatory effects of progesterone and its synthetic derivatives in the NTS may become apparent following downstream transcriptional changes of target genes, such as *Phox2b* and *Phox2a*, as shown here, or through yet-unidentified pathways. Since the functional and transcriptional role of PHOX2B on cellular function in these NTS neurons is unknown, it will be an important step forward to understand what role PHOX2B may have on the respiratory relay network, beyond its key role in the development of the autonomic nervous system and the NTS. Interestingly, recent work shows that PHOX2B neurons in the NTS are chemosensitive and partial lesioning of PHOX2B NTS neurons reduces the chemoreflex response [71,72], while their stimulation potentiates breathing [72], suggesting that these neurons may potentially contribute to chemoreflex responses in physiological conditions or in the absence of a functional RTN.

It is well known that CCHS is caused by mutations in the *PHOX2B* gene, resulting in aberrant protein production [73,74,75,76], although the exact pathogenetic role of the PHOX2B mutant protein is still unclear. Many mechanisms have been proposed for the insurgence of CCHS, among which haploinsufficiency is one [77]. However, respiratory defects may also depend on the gained toxic function by mutant proteins [74,78,79]. Given the role of PHOX2B as a fundamental transcriptional regulator, it is reasonable to assume that transcriptional dysregulation, resulting in abnormal expression of target genes, may be an important pathogenetic mechanism. Given the ETO-induced changes observed in the NTS, it will be important to investigate what is the transcriptional role of PHOX2B in these neurons and whether the down-regulation of PHOX2B protein expression may affect the expression of altered molecular targets in pathological conditions.

## 4. Materials and Methods

### 4.1. Experimental Animals

Experiments were performed using female Sprague–Dawley rats (age 10–20 weeks, mean ± SD: 14 ± 2 weeks) bred born to pregnant dams obtained from Charles River and housed at the University of Alberta Health Sciences Animal Housing Facility to (Senneville, QC). Animals were maintained on a 12-h dark/light cycle and received food and water *ad libitum*. All handling and experimental procedures were approved by the Health Science Animal Policy and Welfare Committee at the University of Alberta (AUP#461) and performed in accordance with guidelines established by the Canadian Council on Animal Care.

### 4.2. Determination of Oestrous Cycle

Vaginal smears by lavage were obtained daily to monitor changes in vaginal cytology resulting from fluctuations in circulating hormones over the oestrous cycle. Smears were obtained using a plastic pipette filled with ~100 µL distilled water and examined under a light microscope (Zeiss, Primo Star, Jena, Germany). Proestrus was defined by the disappearance of leukocytes from the preceding day with a dominance of nucleated epithelial cells either appearing in clumps or with mucous, as described by [80]. Only rats exhibiting regular oestrous cycles of 4–5 days were utilized in experiments.

### 4.3. Surgical Implantation of Nexplanon^®^

Nexplanon^®^ rods (MERCK, Kenilworth, NJ, USA) were implanted subcutaneously to deliver Etonogestrel (ETO). In an initial pilot study, 14 female rats (200–250 g) were implanted with either 250, 500 or 1000 μm long sections of Nexplanon^®^ rods to determine the appropriate length to achieve comparable ETO serum levels as observed in women instrumented with Nexplanon^®^ rods for contraception (~200 pg/mL [40,41]). Analysis determined that 1000-µm length of Nexplanon^®^ rod resulted in the desired serum levels and was used in all subsequent experimental rats. Briefly, rats were pre-treated with 1.5 mg/kg Metacam analgesic and anesthetised with isoflurane (3% in 21% O_2_; via nose cone) and a 0.5 cm skin incision was made below the shoulder blades (using aseptic techniques) to permit implantation of the Nexplanon^®^ rod (1000 µm; SHAM rats received the same surgical procedures but no implant). Bupivacaine (0.1 mL, s.c.) was administered post-surgically. Four weeks following implantation, rats were anesthetized with urethane (1.5 g/kg, i.p.) and ~2 to 2.5 mL blood samples were collected intracardially. After 30 min at room temperature, samples were centrifuged at 2000× *g* (15 min) and the serum stored at −20 °C. Serum ETO concentration was determined by liquid–liquid extraction methods performed by Q2 lab solution (Q2 Solutions, New York, NY, USA). Both serum and the internal standards underwent reverse phase high-performance liquid chromatography and tandem mass spectrometric detection (SCIEX, API6500, Framingham, MA, USA) utilizing a turbo ion spray interface in the positive ion mode.

### 4.4. Data Acquisition and Analysis of Respiratory Measurements

Rats were habituated to the whole-body plethysmography chambers (Buxco, 5 L) on two occasions (~1.5 h) 3–4 days before baseline recordings. On the day of the experiment, rats were placed in the chamber and testing commenced once the animals were quiet but awake (30 min–1 h). Various gas mixtures were produced by a GSM−3 (CWE Inc., Ardmore, PA, USA) and delivered continuously at 1.5 L/min. Ventilatory reflexes were measured during exposure to 5% and 7% CO_2_, and 10% O_2_ (8–15 min). Oxygen consumption and CO_2_ production were determined by recording the% composition of gasses flowing into, and out of, the recording chamber (Respiratory Gas Analyzer, AD Instruments). Estrous testing conducted post-experimentally in ETO-treated rats verified a drug-induced cessation of oestrous cycling, resulting in maintenance of the dioestrus and/or proestrus stages. SHAM rats were recorded only in the proestrus stage to limit confounding effects of cycling sex hormones on breathing.

Respiratory data were analyzed from rats during quiet wakefulness in the last 5 min period of each gas composition. We used the barometric method (open-flow system) described by [81,82]. Briefly, raw pressure signals were recorded by a Validyne differential pressure transducer connected to a CD15 carrier demodulator (Validyne Engineering, Northridge, CA, USA) and digitized using a Powerlab 8/35 (AD Instruments, Sydney, Australia). Analysis was done in LabChart 8 (v8.1.19, AD Instruments, Colorado Springs, CO, USA) using the Blood Pressure module to derive tidal volume (T_V_) and breathing frequency (*f*, breaths·min^−1^). The amplitude of the pressure signal was converted to T_V_ (ml·kg^−1^) using the equations of [83] and calibrated against 1 mL of dry air injected into the empty chamber using a rodent ventilator (*f* = 50, 75, 100, 150 beats·min^−1^). Chamber temperature and humidity were continuously monitored using a RH-300 water vapour analyzer (Sable Systems, Las Vegas, NV, USA) plus HPR Plus Handheld PIT Tag reader (Biomark, Boise, ID, USA) or digital thermometer/hygrometer sensor (Wifehelper), depending on the setup configuration. Rectal temperature was read using a Homeothermic Monitor with Probe (507222F, Harvard Apparatus, Holliston, MA, USA). Minute ventilation ($\overset{•}{V}$_E_) was calculated as *f* × T_V_ and expressed as ml·min^−1^·kg^−1^, and allometrically corrected for weight gain according to [67] resulting in the parameter, $\overset{•}{V}$_E_ ALLO. Metabolic parameters $\overset{•}{V}$O_2_, $\overset{•}{V}$CO_2_, $\overset{•}{V}$_E_/$\overset{•}{V}$O_2_ and $\overset{•}{V}$_E_/$\overset{•}{V}$CO_2_ were calculated by pull-mode indirect calorimetry [67]. Briefly, the composition of dry gas flowing into, and out of, the recording chamber was measured by using an AD Instruments Gas Analyzer (Colorado Springs, CO, USA) and applying the equations of [84,85]. Metabolic measurements were corrected to standard temperature and pressure (STPD). Breath cycle period was measured between the start of inspiration to the subsequent inspiration using the peak analysis feature in Labchart 8 applied to 10 min of room air data. Quantification of Poincaré plot data was performed using equations defined by [86,87]. Briefly, standard deviation calculated from the dataset defines the width (SD1) and length (SD2) of a fitted ellipse (Figure 6A, inset). Area is calculated as π × SD1 × SD2. Data were analysed using SPSS (13.0) to perform a student’s *t*-test (weight gain) or Mann–Whitney test (body temperature; Figure 5B) or repeated-measures ANOVA with Bonferroni post-hoc (Figure 5A,C–F and Figure 6).

### 4.5. Tissue Collection and Thionine Staining

Rats were anesthetized with 3% isoflurane (nose cone) and blood samples collected intracardially and the serum stored at −80 °C until further use. Under isoflurane anaesthesia, rats were then decapitated, and the brain was quickly removed and placed on an ice-cold tissue chopper (Mc Ilwain Tissue Chopper). The brain was sliced into 1–2 mm sections and regions of interest were collected using a 2 mm diameter tissue punch according to gross anatomical coordinates: medial hypothalamus (MH; 2 mm section taken 2 mm caudal to anterior commissure, posterior; either side of 3rd ventricle), locus coeruleus (LC; 1 mm section taken 1 mm caudal to the aqueduct end; left and right sides of 4th ventricle). The parafacial region (PF), including the retrotrapezoid nucleus and midline structures, was collected as the 1 mm ventral margin of the brainstem in a 1–1.5 mm slice caudal to the one used for LC. The dorsal vagal complex (DVC), including NTS, AP and X, was collected from the dorsomedial brainstem in a 2-mm thick section collected around the obex. Fresh resected tissues were immediately frozen in dry ice and stored at −80 °C for RNA and protein extraction.

To confirm the anatomical boundaries and reduce the variability among samples, the leftover tissues were processed for thionin counterstaining. After dissection, tissue was fixed in PFA, cryoprotected in 30% sucrose and frozen in Tissue-Tek O.C.T compound at −80 °C, and sectioned on a cryostat (MODEL CM1950, Leica Biosystems, Buffalo grove, IL). Fifty µm thick brain sections were washed in phosphate buffer saline (1X PBS: 11.9 mM phosphates, 137 mM sodium chloride, 2.7 mM potassium chloride), mounted sequentially on microscope slides, and dried overnight. Slides were then rehydrated in increasingly graded ethanol and reacted with thionin for 30 s, dehydrated, and coverslipped with CytoSeal 60 mounting medium (Electron Microscopy Sciences) and observed under Zeiss Primo Star binocular microscope.

### 4.6. Total RNA and Protein Extraction

The total RNA and proteins were isolated from the same tissue simultaneously. Tissue samples were thawed, disrupted and homogenised in QIAzol Lysis Reagent (Qiagen). After the addition of chloroform, the homogenate was separated into aqueous and organic phases by centrifugation (6000× *g*). The RNA in the upper phase was collected and purified using the RNeasy Mini kit Qiagen), according to the manufacturer’s instructions.

Proteins in the lower organic phase were precipitated by adding 4 volumes of ice-cold acetone and incubated for 30 min on ice. After centrifugation at 1200× *g*, the pellet was washed with ice-cold ethanol and protein samples from three rats were pooled together. Finally, pooled proteins were resuspended in sodium dodecyl sulphate (SDS) buffer and stored at −80 °C; the total protein concentration was evaluated using BCA Protein Assay (Thermo-Fisher Scientific, Waltham, MA, USA).

### 4.7. Reverse Transcription and qPCR

To study the effect of ETO on *Phox2b* and its target genes mRNA expression level, one µg of RNA was reverse transcribed using the GoScript™ Reverse Transcriptase kit (Promega, Madison, WI, USA) according to the manufacturer’s instruction manual, and the cDNAs of interest were then amplified and quantitatively analysed using a StepOnePlus™ Real-Time PCR System (Thermo-Fisher Scientific, Waltham, MA, USA). The TaqMan^®^ primer and probe assays (Life Technologies, Inc.) used in the study were the following: rat Phox2b (ID #Rn01413076_mH), rat Phox2a (ID #Rn00587898_m1), rat Pgr (ID #Rn01448227_m1), rat Th(ID #Rn00562500_m1), rat Dbh (ID #Rn00565819_m1) and the endogenous control rat Actb (ID #Rn00667869_m1). Each sample was run in duplicate, and the results were calculated using the 2^−Δ^^Ct^ and the 2^−ΔΔ^^Ct^ methods to allow for normalization of each sample to Actb [88]. Results are shown as relative expression ± SD, and statistical analysis was performed to assess differences, within each brain area, between SHAM and ETO groups (unpaired two-tail student’s *t*-test) using GraphPad Prism 5 Software (GraphPad Software, Inc., San Diego, CA, USA). *p* values < 0.05 were considered significant.

### 4.8. Western Blot Analyses

In order to evaluate the effect of ETO on PHOX2B and its target gene protein levels, 80 μg of protein extract from the areas of interest was analysed by means of Western blot as previously described [89]. Protein extracts were separated on a 10% denaturing polyacrylamide gel in the presence of 0.1% SDS extract. A standard molecular weight (Precision Plus Protein™ Standards Bio-Rad) was loaded in parallel to assess protein molecular weights. After gel separation at 80 V in Tris-glycine (Trizma base 25 mM, Glycine 192 mM, SDS 0.1%), the proteins were transferred to nitrocellulose membranes (Amersham) by electroblotting at 110 mA 90 min in transfer buffer (Trizma base 25 mM, Glycine 192 mM, MetOH 20%). The membrane was then washed with distilled H_2_O, saturated with blocking buffer (non-fat milk 5%, Tris-HCl pH 7.5 20 mM, NaCl 150 mM, Tween^®^20 0.1%) for at least 1 h and incubated over night at 4 °C with the appropriate antibody diluted in blocking buffer. The primary antibodies used in the study were: polyclonal rabbit anti-PHOX2B (1:500 Novus Biologicals), monoclonal rabbit anti-beta Tubulin SR25-04 (1:1000; Novus Biologicals) and polyclonal anti-TH (1:1000; Millipore Sigma). On the following day, the membrane was washed in blocking buffer (3 × 5 min), incubated with the secondary antibody, and diluted in blocking solution for 1 h. The excess antibody was then removed by multiple washes with blocking buffer; TS 1X buffer (Tris-HCl pH 7.520 mM, NaCl 150 mM) + Tween^®^20 0.1%; three washes in TS 1X + Tween^®^20 0.3% and three final washes in TS 1X. Primary antibodies were revealed by infrared-conjugated anti-rabbit IgG IRDye^®^ (800CW, Abcam, Cambridge, UK) and scanned with the Odyssey Infrared Imaging System (LI-COR Biosciences, Lincoln, NE, USA). Densitometric analysis of the obtained signals was carried out using Image Studio software (LI-COR Biosciences, Lincoln, NE, USA), and the results are shown as the mean ± SD of at least three independent experiments. Statistical analysis was performed to assess differences, within each brain area, between SHAM and ETO groups (two-tailed student’s *t*-test; GraphPad Prism 5). *p* values of < 0.05 were considered significant.

### 4.9. In Situ Hybridization (RNAScope) and Immunofluorescence

Following the plethysmography recordings (4-weeks post-Nexplanon implantation), rats were transcardially perfused in 4% paraformaldehyde (PFA) and the brains were post-fixed in 4% PFA and cryoprotected in 30% sucrose in 1× PBS frozen in O.C.T and sectioned on a cryostat (MODEL CM1950, Leica Biosystems, Buffalo Grove, IL, USA). Thirty µm thick sections were mounted on slides for combined RNAScope^®^ in situ hybridization (Advanced Cell Diagnositcs-ACD Bio, Newark, CA, USA) and immunofluorescence detection to quantify *Phox2b* mRNA and protein expression within the RTN and the DVC according to our previous protocols [90]. In order to reduce inter-animal variability in our results, the experiments were performed concurrently on all tissue sections by an investigator blinded to the animal group.

For the RNAScope assay, slides were processed as detailed in [90] and incubated with probes for *Phox2b* (Rn-Phox2b-O1-C1 #1064121-C1, ACDBio, Newark, CA, USA) and *Nmb* (RN-NMB-C2 #494791-C2, ACDBio) for 2 h at 40 °C. In parallel, two sections/rat were treated with positive (low-copy housekeeping gene) and negative (non-specific bacterial gene) control probes provided by ACDBio. Finally, slides were processed using the RNAScope Multiplex Fluorescent Assay kit V2 (ACDBio) according to the manufacturer’s instructions. *Phox2b* and *Nmb* probes were visualized using Opal 570 and Opal 690 reagent, respectively (1:1000; PerkinElmer, Woodbridge, ON, USA).

Immediately following the RNAScope assay, immunofluorescence staining was performed using commercial mouse monoclonal antibody against PHOX2B (B-11: sc-376997, 1:100, Santa Cruz Biotechnology, Dallas, TX, USA) and the goat polyclonal antibody against ChAT (1:500; #AB144P, EMD Millipore, Burlington, MA, USA) followed by the secondary antibodies as follows: donkey CY5-conjugated anti-mouse IgG and donkey Cy2-conjugated donkey anti-goat IgG (1:200; Jackson Immuno Research Laboratories Inc., West Grove, PA, USA). Slides were then cover-slipped with Fluorosave mounting media (EMD Millipore, Burlington, MA, USA) and fluorescence labelling was observed and acquired under a Leica TCS SP8 Laser Scanning Confocal microscope (Concord, ON, USA).

### 4.10. Histological Analysis and Quantification

For quantification of *Pho2xb* mRNA and protein expression in serial section, the opening of the central canal was set as our reference point (−13.50 mm from Bregma according to the Paxinos atlas coordinates [91]). To identify and quantify the expression of *Phox2b* in RTN neurons, we analysed one every nine sections (240 µm interval) in 1 mm caudo-rostral distribution from the opening of the central canal and encompassing the facial motor nucleus. The mRNA expression for *Nmb* was used as marker of RTN CO_2_-sensing neurons [92]. To investigate *Phox2b* expression within the DVC we selected 4 sections surrounding the opening of the central canal (i.e., −14.22 µm, −13.74 µm, −13.50 µm−13.26 µm and −12.78 µm from bregma), and ChAT staining was used as anatomical marker to distinguish between PHOX2B-positive cells in the X (ChAT^+^/PHOX2B^+^) and the NTS (ChAT^−^/PHOX2B^+^).

Under the Leica TCS SP8 Laser Scanning Confocal microscope, exposure time and acquisition parameters were set for the SHAM group and kept unchanged for the entire dataset acquisition. The collected images were than analysed using FIJI software [93] by selecting one cell at a time in an image and measuring the area, integrated density and mean grey value. Using the calculation for the corrected total cell fluorescence (CTCF) = integrated density (area of selected cell × mean fluorescence of background reading), as previously described [94], the fluorescence intensity of each cell was calculated using Excel (Microsoft Office 365 for Windows). For each image, three background areas were used to normalize against autofluorescence. For each section three images were acquired with a 25× objective, so that at least fifty cells per single tissue sample were available for analysis. This resulted in a more accurate mean fluorescence value under each condition, which was then used for statistical analyses. The statistical analyses were made using repeated-measures ANOVA and GraphPad Prism 5 Software (GraphPad Software, Inc., San Diego, CA, USA). *p* values of < 0.05 were considered significant.

## Figures and Tables

**Table 1:** Poincaré analysis of breath cycle period variability between ETO-treated rats and SHAM controls. Standard deviation (SD1, SD2) calculated for each rat defines two axes an ellipse fitted to the dataset. Values are expressed as mean ± SD. Statistical significance (*) indicates *p* < 0.05 for Bonferroni post hoc test.

	SHAM		ETO		
Poincaré Analysis	Pre-Surgery	4 Weeks	Pre-Surgery	4 Weeks	*p*-Value
SD1	0.085 ± 0.022	0.086 ± 0.019	0.086 ± 0.016	0.101 ± 0.019	Pre-post *p* = 0.129 Interaction *p* = 0.178 Treatment *p* = 0.305
SD2	0.149 ± 0.026	0.152 ± 0.034	0.151 ± 0.024	0.196 ± 0.043	Pre-post *p* = 0.022 * Interaction *p* = 0.038 * Treatment *p* = 0.080
SD1/SD2	0.569 ± 0.098	0.579 ± 0.132	0.579 ± 0.109	0.531 ± 0.113	Pre-post *p* = 0.547 Interaction *p* = 0.359 Treatment *p* = 0.671
Area	0.041 ± 0.017	0.042 ± 0.016	0.041 ± 0.012	0.063 ± 0.019	Pre-post *p* = 0.019 * Interaction *p* = 0.028 * Treatment *p* = 0.110

## Data Availability

The data presented in this study are available on request from the corresponding author.
